# Supplementary material for: Splice-Junction-Based Mapping of Alternative Isoforms in the Human Proteome
Source: Cell Rep. Author manuscript; Available in PMC 2020 Jan 15. (PMC6961840; doi:10.1016/j.celrep.2019.11.026)
Supplement: 2 [file NIHMS1546469-supplement-2.zip › DF1/PXD010154/Esophagus/Esophagus_24_ILKAP_GPLLFDDLPPASSTDSGTVFR.pdf]

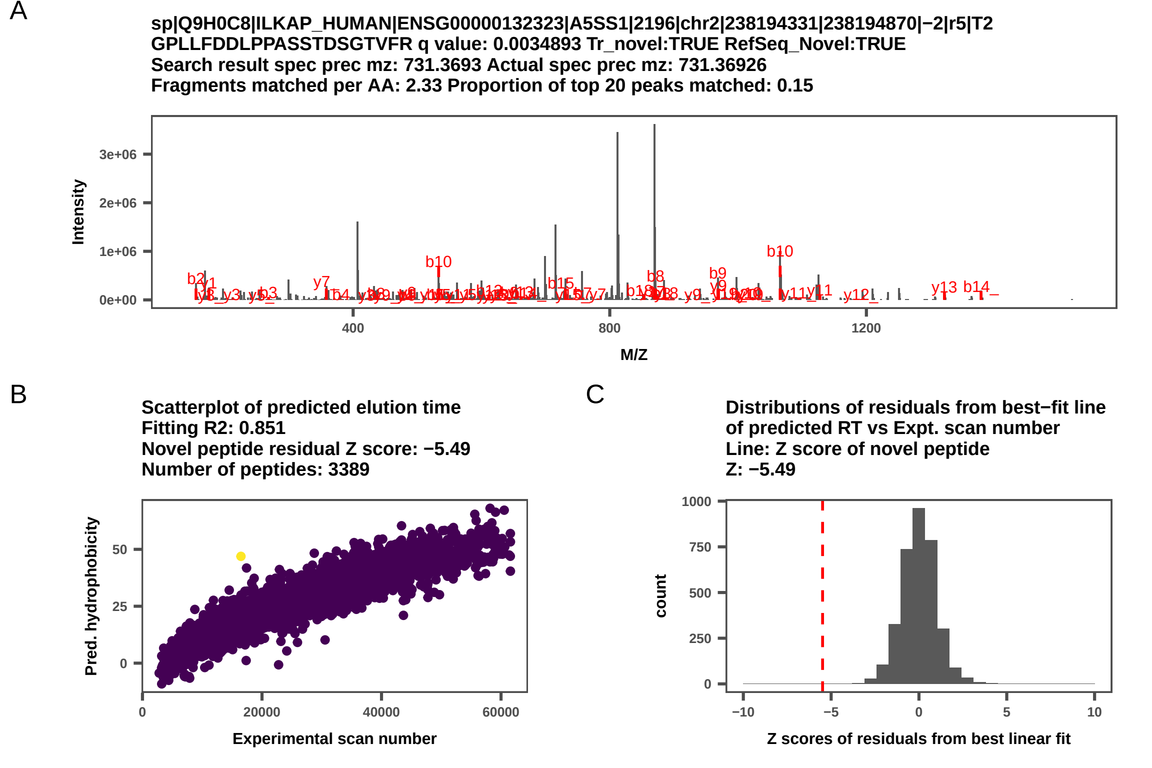
A
sp|Q9H0C8|ILKAP_HUMAN|ENSG00000132323|A5SS1|2196|chr2|238194331|238194870|−2|r5|T2
GPLLFDDLPPASSTDSGTVFR q value: 0.0034893 Tr_novel:TRUE RefSeq_Novel:TRUE
Search result spec prec mz: 731.3693 Actual spec prec mz: 731.36926
Fragments matched per AA: 2.33 Proportion of top 20 peaks matched: 0.15
Intensity
3e+06
2e+06
1e+06
0e+00
b10
b2
y1
y7
b15
b8
b9
y9
b10
y11
y13
b14
400
800
1200
M/Z
B
Scatterplot of predicted elution time
Fitting R2: 0.851
Novel peptide residual Z score: −5.49
Number of peptides: 3389
Pred. hydrophobicity
50
25
0
0
20000
40000
60000
Experimental scan number
C
Distributions of residuals from best−fit line
of predicted RT vs Expt. scan number
Line: Z score of novel peptide
Z: −5.49
count
1000
750
500
250
0
−10
−5
0
5
10
Z scores of residuals from best linear fit
